# Effective disc age: a statistical model for age-dependent and level-specific lumbar disc degeneration using magnetic resonance imaging (MRI)

**DOI:** 10.1007/s00586-025-08729-9

**Published:** 2025-03-01

**Authors:** Harrah R. Newman, John M. Peloquin, Kyle D. Meadows, Barry A. Bodt, Edward J. Vresilovic, Dawn M. Elliott

**Affiliations:** University of Delaware, Newark, USA

**Keywords:** Intervertebral disc, Aging, Degeneration, MRI

## Abstract

**Purpose:**

Intervertebral disc degeneration progresses with normal aging; yet common disc grading schemes do not account for age. Degeneration progression also varies between spine levels and is similarly not accounted for by current grading schemes. These limitations inhibit differentiation between discs with normal and expected aging (non-pathological) and discs with accelerated degeneration (which *may* be pathological). We sought to develop a statistical model to quantify normal age and spine level dependent disc degeneration.

**Methods:**

Eighty-four asymptomatic adult subjects ranging evenly from 18 to 83 years old underwent magnetic resonance imaging (MRI) of the lumbar spine. Subject traits, MRI-derived disc geometry, and MRI biomarkers of T2 relaxation time were evaluated and used to develop a statistical model to predict *effective disc age*, the age at which normal aging would produce a disc’s observed phenotype.

**Results:**

After evaluating several models, a 4-predictor model utilizing 1) subject height, 2) nucleus pulposus T2 relaxation time, 3) disc mid-sagittal area and 4) disc 3D volume, optimally estimated *effective disc age*. The effective age closely tracked true age for spine levels L1-L5 (R^2^ ≈ 0.7, RMSE ≈ 10 years) and moderately tracked true age for L5-S1 (R^2^ = 0.4, RMSE = 14 years). The uncertainty in the effective disc age prediction was ± 3 years as assessed by fivefold cross validation.

**Conclusion:**

We offer a data-driven, quantitative tool to quantify normal, expected intervertebral disc aging. This effective age model allows future research to target discs with accelerated degeneration.

**Supplementary Information:**

The online version contains supplementary material available at 10.1007/s00586-025-08729-9.

## Introduction

Assessment of disc degeneration is an important part of efforts to understand and treat low back pain. Accordingly, many degeneration grading schemes can be found in the literature [1–5]. However, the concept of “disc degeneration” combines many processes and phenotypes. Degenerative changes may be caused by mechanical injury, genetics, environmental factors, or simply normal aging [6–8]. Multiple etiologies may affect a disc simultaneously. Currently, a critical problem in low back pain etiology is an inability to distinguish pathological from non-pathological disc degeneration. Although some weak associations have been reported between degeneration and low back pain [9, 10], most disc degeneration is asymptomatic and of little clinical concern [11–13]. This problem is in part due to an inability to distinguish age-related degeneration (“normal aging”), which is ubiquitous, from all other forms of degeneration [7].

To support hypothesis testing, diagnosis, and treatment, a critical need exists to isolate and quantify expected degenerative changes associated with normal aging. In an effort to remove normal aging from the definition of disc degeneration, degeneration has been redefined as cell-mediated, progressive structural failure combined with “accelerated or advanced signs of aging” [6]. We subsequently refer to such degeneration as *accelerated degeneration*. However, there is currently no tool to quantify whether a disc has normal or accelerated/advanced signs of aging. As the prevalence of lumbar disc degenerative features increases with age, and the prevalence of each feature increases at different rates [7], it may be feasible to quantify normal aging and thus accelerated degeneration of lumbar discs in a data-driven manner.

Currently, disc degeneration is primarily assessed using qualitative grading schemes based on features from magnetic resonance imaging (MRI) such as disc height, signal intensity, and distinction between the annulus fibrosus (AF) and nucleus pulposus (NP) [1, 2, 4]. It is widely acknowledged that these grading schemes are limited by being subjective, qualitative, ordinal [14, 15], prone to adjacent disc bias, and they correlate poorly with pathology [9–11, 16–20]. As a quantitative alternative that is objective and continuous, MRI relaxation times (T2 or T1rho) in the NP have been used during the last decade as biomarkers of disc degeneration [5, 21] and T2 histogram-mapping has shown promise [22]. Numerous studies using these approaches have shown that degenerative grade increases with age [5, 8, 9, 23], and that lower lumbar levels (L4-S1) have higher grades and faster degeneration rates than upper levels [8, 9, 11, 23, 24]. NP T2 is also dependent on both age and spinal level [9, 13, 14]. Importantly, controlling for age and disc level has been shown to strengthen the association of Pfirrmann grade with low back pain [23]. Thus, while associations of degeneration with age and spinal level have been demonstrated, no degeneration schemes have directly accounted for subject age or spinal level. A quantitative grading scheme that directly accounts for age and disc level would therefore be beneficial and is a natural next step.

The objective of this work was to develop a disc degeneration assessment tool that is objective, quantitative, continuous, and accounts for subject age and spine level, such that it would distinguish normal, non-pathological aging from other degenerative changes. Subject traits, MRI-derived disc geometry, and MRI biomarkers can be obtained non-invasively and, individually, have exhibited relationships with disc degeneration and age in prior work [8, 9, 11, 14]. We therefore developed a statistical model based on these predictor variables, using data from 84 asymptomatic subjects of age 18–83 years, to *predict* lumbar disc age. The predicted disc age, subsequently called *effective disc age*, is the age that would be expected, on average, for the disc’s observed phenotype. It is the age a disc *should* have if all its degenerative changes were from normal aging. The difference between the effective disc age and the subject’s true age therefore provides an objective assessment of whether that disc is affected by accelerated degeneration. Consistent with the prevailing consensus that non-pathological degenerative changes are largely due to normal aging, we hypothesized a strong correlation between effective disc age and subject’s true age.

## Methods

### Subject population

A total of 84 subjects (18–83 years old) were assessed, with IRB approval and informed consent. Subjects were evenly distributed across sex and age, such that there were 7 subjects of each sex per age decade (18–29, 30–39, 40–49, 50–59, 60–69, 70–83 years old). This is a larger and better-balanced data set than used in the development of prior lumbar disc degeneration grading schemes [1, 2, 4, 5]. Study inclusion criteria were that the subjects never had back surgery, chronic low back pain (> 3 months duration), or acute low back pain in the last 2 years, and had no conditions that would inhibit their safety in magnetic resonance imaging (MRI). While there may be some recall bias for prior pain, this is unlikely given the duration of chronic pain and the recent criteria for acute pain, moreover, similar criteria are used in other studies [13, 16, 25, 26]. Subjects needed to score under 20% on an adapted Oswestry Disability Index, indicating no to minimal disability from low back pain [27, 28]. All subjects’ index scores were well below this threshold, at 0.3% ± 1.6%. The use of asymptomatic subjects supported the objective of quantifying the effects of *normal* aging via the effective disc age model. Subjects were recruited with the support of the University’s Center for Human Research Coordination and met race distribution consistent with our region (White 79%, Asian 12%, Black 6%, More than one race 3%).

### Study protocol

Subjects were asked to minimize their activity prior to the MRI appointment. Subjects arrived at the MRI facility early in the morning (~ 7AM) and lay supine for at least 45 min to unload the spine from any unavoidable morning activity. Following spine unloading, subjects were scanned in a supine position with head/neck supported by a pillow and legs supported by a foam wedge bolster. The same pillow/bolster were used for all subjects.

### MRI protocol

**Fig. 1:**
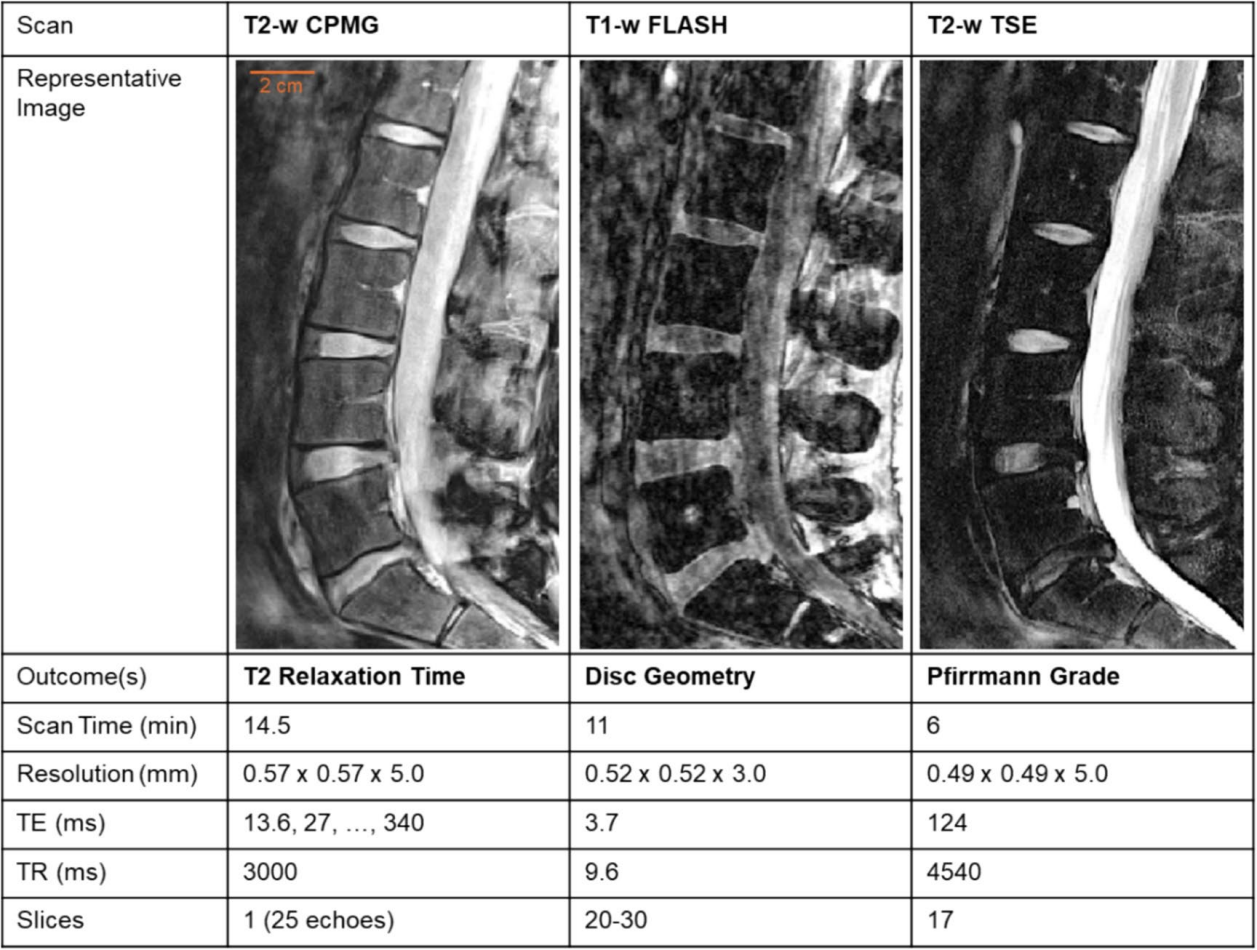
Magnetic resonance imaging (MRI) scan sequences and representative images. The T2-w CPMG scan was conducted on the mid-sagittal slice over 25 echoes of sequential multiples of 13.6 ms, up to 340 ms. The T1-w FLASH captured the entire disc volume, such that subjects with larger spines or greater curvature required more slices. The T2-w TSE was used to evaluate Pfirrmann grade

All MRI was conducted with a Siemens Magnetom Prisma 3T whole-body scanner. The lumbar spine discs (L1-S1) were assessed with three sagittal MRI scans [27] (Fig. 1): 1) a single, mid-sagittal slice T2-weighted Carr-Purcell Meiboom-Gill (T2-w CPMG) scan to measure T2 relaxation time, 2) a 3D T1-weighted Fast Low Angle Shot (T1-w FLASH) scan to measure disc geometry, and 3) a 3D T2-weighted Turbo Spin Echo (T2-w TSE) scan to assess disc degeneration by Pfirrmann grade.

### Data analysis

#### T2 relaxation time

**Fig. 2:**
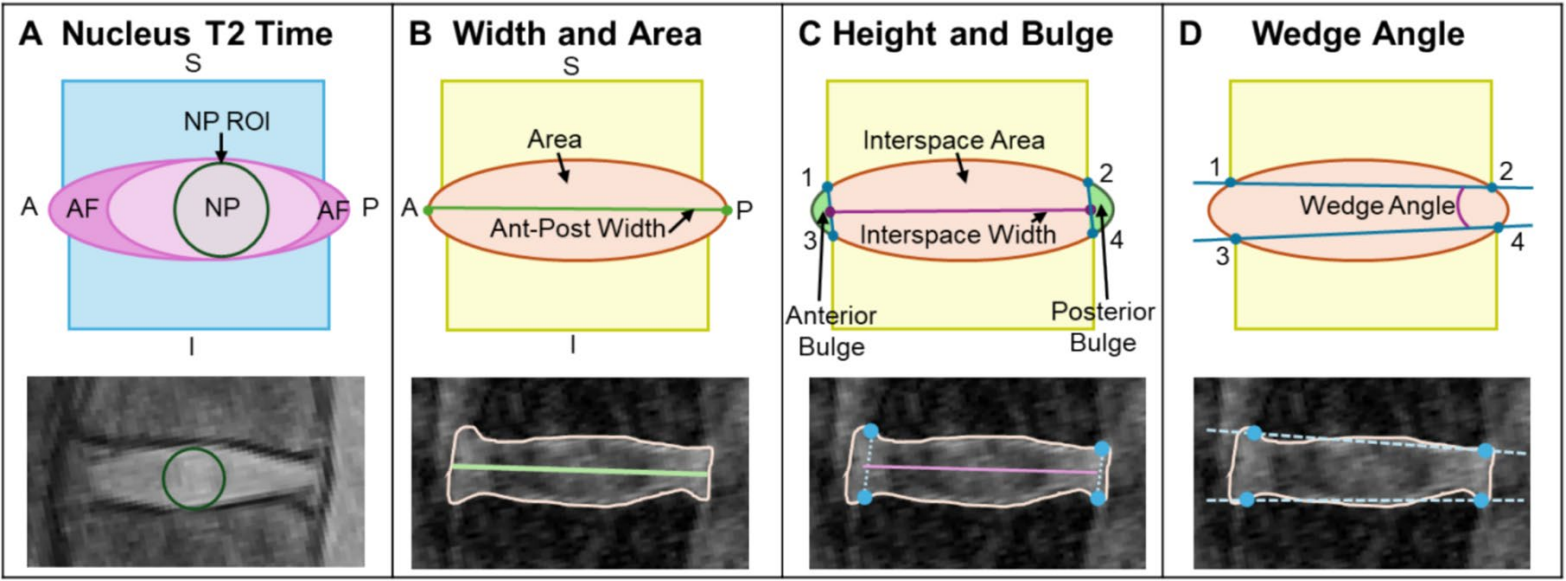
MRI outcome measures. **A** T2 relaxation time was calculated from the nucleus pulposus region of interest (NP ROI). Disc geometry was quantified by **B** disc mid-sagittal area, anterior–posterior width, **C** disc height, anterior and posterior disc bulge, and **D** disc wedge angle

The T2 relaxation time is an MRI biomarker that is highly correlated with tissue water content. T2 time was assessed from the T2-w CPMG mid-sagittal scan by two methods: a circular region of interest (ROI) in the nucleus pulposus (NP) and by region along an anterior–posterior line. Firstly, the T2 time was evaluated from the intensity decay of a circular ROI, selected to be as large as possible but encompassing only the NP, typically 80–120 voxels (Fig. 2A) [27, 29]. The intensity decay was fit with a noise-corrected, single term exponential fit [27, 29]. Secondly, regional T2 time was assessed across five regions: the anterior AF (AAF), anterior transition (Atrans), NP, posterior transition (Ptrans), and posterior AF (PAF). Regional T2 analysis is described in detail in the supplemental text and figures.

#### Disc geometry

Discs were manually segmented using ITK-SNAP [27, 30]. Disc volume was calculated as the segmented voxel quantity multiplied by the unit voxel volume. All subsequent disc geometry quantifications were done exclusively in the mid-sagittal image slice. Disc area was calculated as the segmented voxels in the mid-sagittal slice multiplied by the unit voxel area (Fig. 2B). Disc width was calculated as the anterior–posterior length across the disc at mid-height (Fig. 2B). The disc height was calculated as the disc interspace area divided by the interspace width, where the interspace was defined by manually placed fiducial markers on the adjacent vertebral bodies (Fig. 2C). The anterior and posterior disc bulges were quantified as the areas outside of the interspace markers (Fig. 2C). Disc wedge angle was measured as the angle between the superior and inferior line segments formed by the vertebral body fiducial markers (Fig. 2D).

#### Pfirrmann grade

The Pfirrmann grade, which evaluates disc health on a scale from 1 (healthy) to 5 (degenerated) was assessed from the T2-w TSE scan [2]. The discs were analyzed by three trained graders who reached consensus on each disc’s grade.

### Statistical analysis of measured parameters

Disc geometry and MRI biomarkers (Table 1) were evaluated for significant differences between spine levels and age groups (grouped by decade) using a linear mixed model with the following effects: spine level (5 levels, fixed effect), age decade (6 groups, fixed effect), and subject ID (random effect). Subject sex was initially considered as a fixed effect, but had no significant effect, in agreement with other’s findings [8], and was therefore excluded. A Tukey HSD test was used to assess differences between spine levels. The effect of aging on MRI measurements and Pfirrmann grade was evaluated by linear regression with age, by spine level. All analyses were conducted in JMP statistical software (JMP Pro 17, SAS Institute Inc).

**Table 1:**
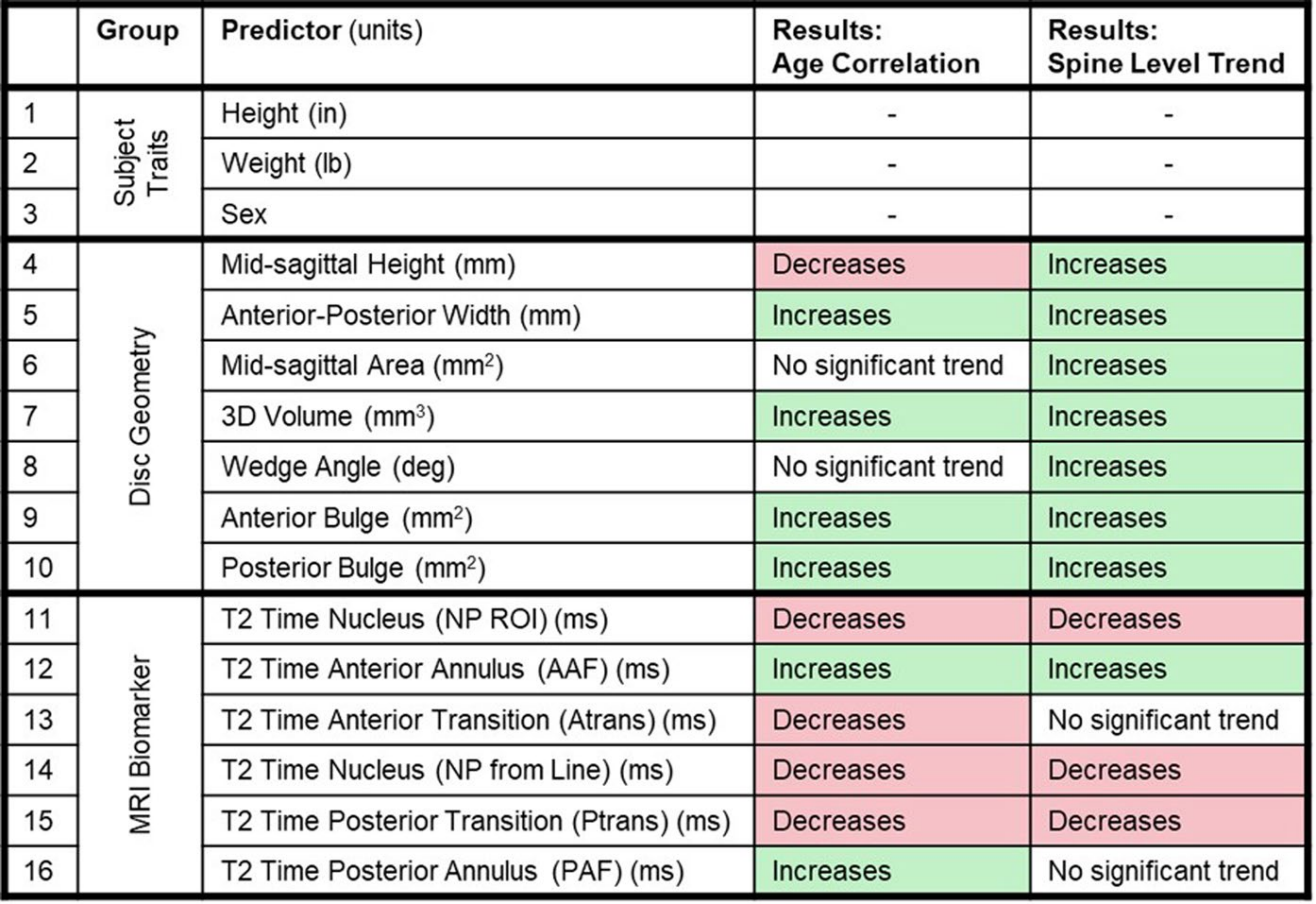
Potential predictors for the effective disc age included subject traits, disc geometry, and MRI biomarkers. Disc geometry and MRI biomarkers change with both subject age (green for increasing; red for decreasing) and spine level (green for inferiorly increasing, red for inferiorly decreasing)

### Effective disc age statistical model

We sought to create a model to calculate a disc’s “effective age” where we would expect a disc with normal aging would have an effective disc age = subject age and a disc with accelerated degeneration to have effective disc age > subject age. We developed the model with data from an asymptomatic population, expected to represent normal aging changes.

Sixteen potential predictors including subject traits, disc geometry, and MRI biomarkers (Table 1) were tested for their ability to predict the subject’s true age using linear regression. Each spine level was treated independently. To support this decision, we tested for intra-individual dependencies, which were either weak or not present. Only disc-specific measures (i.e., disc wedge angle), rather than lumbar spine measures (i.e., Cobb angle) were considered as model predictors. All analyses were conducted in JMP statistical software.

#### Predictor selection

Given the large number of candidate predictors for disc age (Table 1), all possible subsets regression was conducted to select sets of 1-, 2-, 4-, 6-, and 8- predictors, using goodness of fit (R^2^) and root mean square error (RMSE) as the model evaluation criteria. The intent was to choose a parsimonious set of predictors—the fewest predictors possible to achieve good fit and avoid overfitting. All possible subsets regression was initially conducted for each spinal level individually, but the sets of selected predictors were similar across levels, so the final selection considered all discs simultaneously.

#### Model selection

A separate linear regression model for disc age was then fit for each of the 1-, 2-, 4-, 6-, and 8- predictor sets. The model intercept and regression coefficients were fit independently for each spine level, as many of the predictors had different relationships with age between levels. A sixth model was also created using all 16 predictors to provide a baseline of comparison. Model residuals were evaluated for normality, homogeneity of variance, the presence of outliers, and the explained response variation. The most parsimonious model that retained prediction performance (insignificant improvements to R^2^ and RMSE from adding more predictors) was selected as the final effective disc age model.

#### Model evaluation

The final model was evaluated by comparing the predicted ages from the model (the “effective disc age”) to the subjects’ true age. Given the subjects were all asymptomatic, we expected a strong correlation between the effective disc age and subjects’ true age. We evaluated the contribution of predictors using standard beta coefficients, predictor contribution uniqueness using variance inflation factors, and predictor significance using probability values (*p* values) for each spine level.

Lastly, the effective disc age model was evaluated by a fivefold cross validation, where for each ‘fold’ the model was created with 80% of the data and then validated on the remaining 20%. This process was repeated 5 times such that each disc was in the model development group 4 times and the validation group 1 time. The model uncertainty was determined from the range of RMSE across the 5 cross validation models for each disc level. The model was ultimately deemed validated and generalizable if the model uncertainty (from cross validation sets) was less than the actual model (from all data) RMSE.

## Results

### Parameter outcomes

**Fig. 3:**
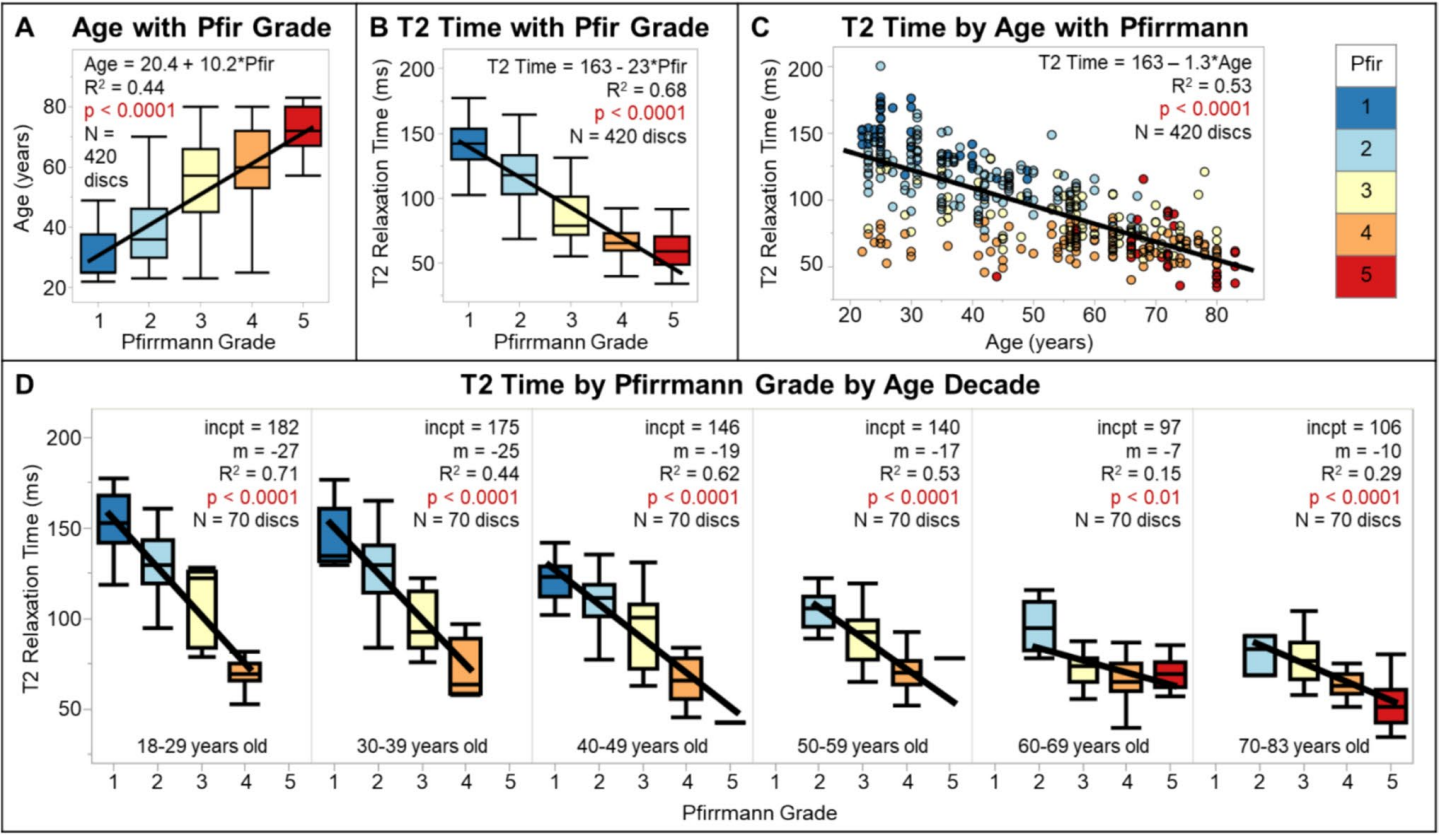
Pfirrmann grade is significantly correlated with **A** subject age and **B** nucleus T2 relaxation time. Furthermore, the relationship between Pfirrmann grade and T2 time changes with age. **C** Young discs that have low T2 time also have high Pfirrmann grades, but all older discs have low T2 time and mostly high Pfirrmann grades. **D** The association between T2 relaxation time and Pfirrmann grade diminishes with age

Disc height decreased with increasing subject age, while disc width, volume, and bulge increased with increasing subject age (Table 1, Fig. S3), however, these correlations of disc geometry with age had low R^2^ values. All disc geometry measures increased from the superior to inferior spine levels (Table 1, Fig. S2). MRI biomarker T2 time in the NP and NP-AF transition regions decreased with increasing age and inferior spine level (Table 1; Figs. 3C, S4). The T2 time in the AF increased with increasing subject age (Table 1; Fig. S5).

Pfirrmann grade correlated with age and NP T2 time, as expected (Fig. 3A, B and C). The relationship between Pfirrmann grade and T2 time changed with subject age. When this correlation is visualized by decade (Fig. 3D), the decreased intercept and slope seen with increasing age decade indicates the large impact age has on both T2 time and Pfirrmann grade, which demonstrates the need for an age-dependent degeneration assessment, as developed in this study.

### Effective disc age model

#### Predictor and model selection

The NP T2 time was the most predictive single parameter for estimating effective disc age from subset regression and was included in all candidate models (Table 2). The 2-predictor model added disc width, while the 4-predictor model added subject height, disc area, and disc volume. The additions for the 6- and 8-predictor models are in Table 2.

**Table 2:**
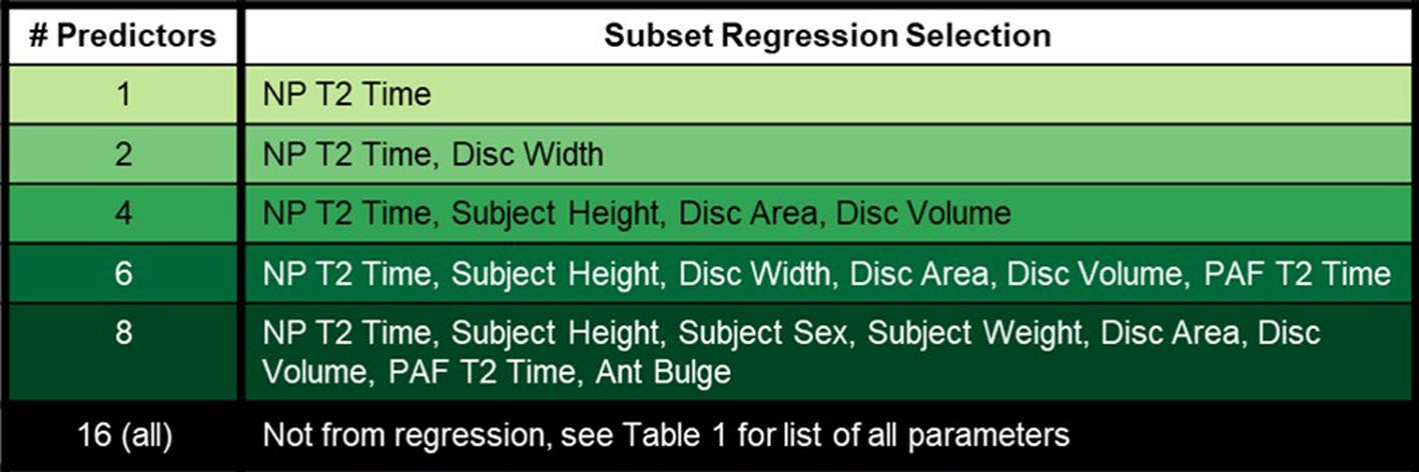
Subset regression recommended models for 1-, 2-, 4-, 6-. and 8-predictor model fits. A best possible model including all 16 available predictors was also considered

**Fig. 4:**
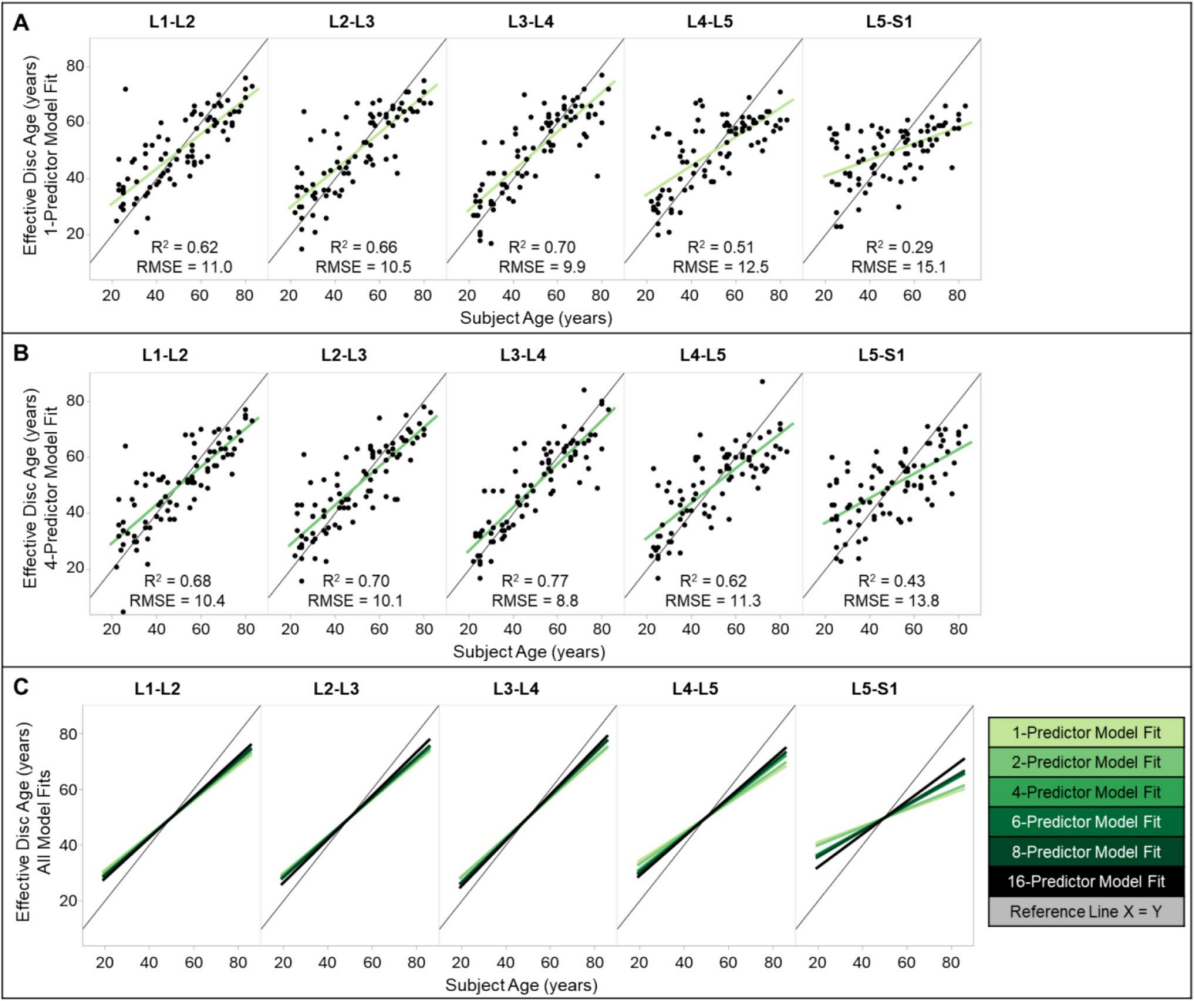
A Comparison of subject true age to the calculated effective disc age using **A** the 1-predictor model, **B** 4-predictor model, and **C** all models by spinal level

**Fig. 5:**
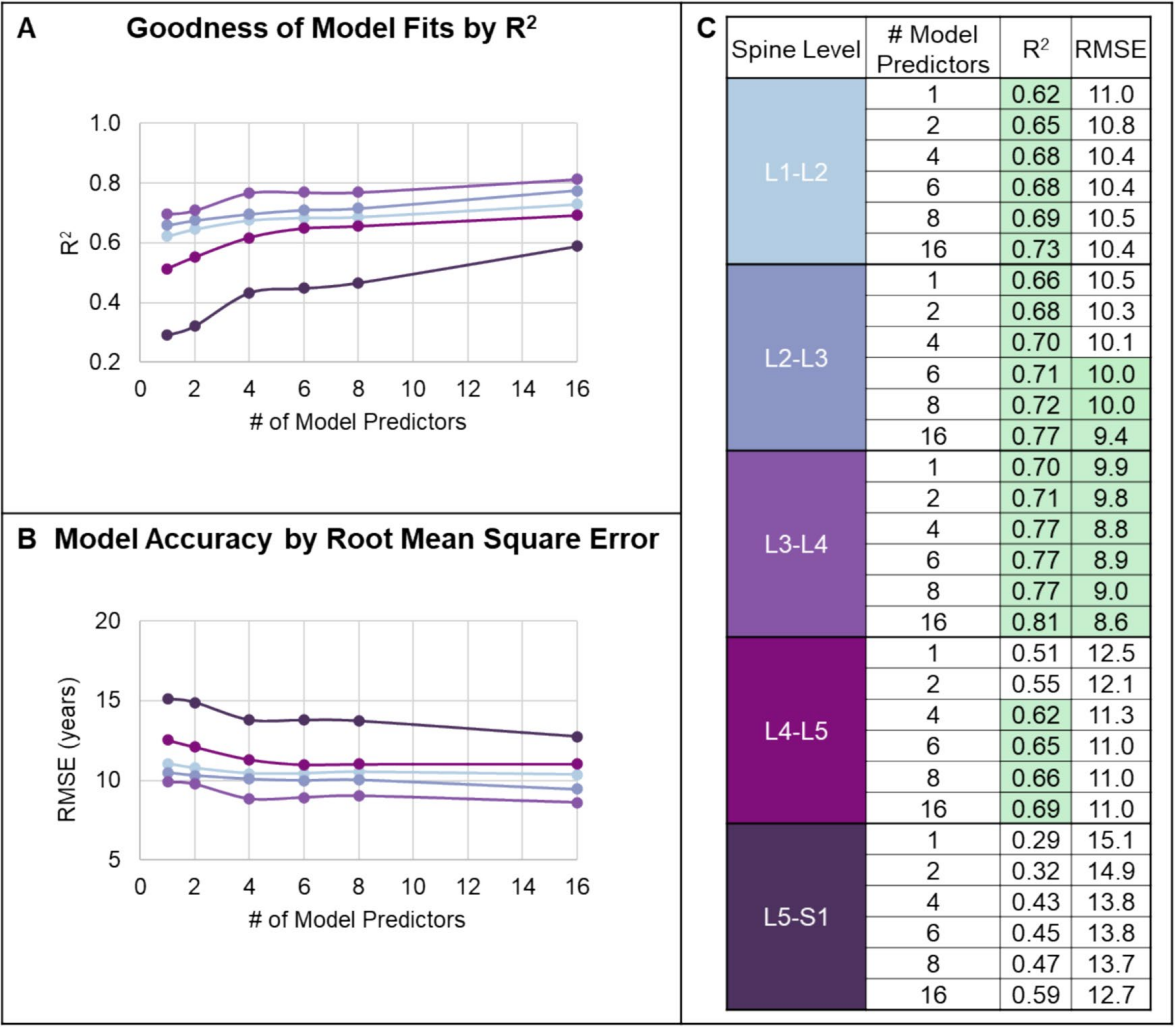
Model fits were evaluated by spine level for **A** goodness of fit R^2^ and **B** accuracy by root mean square error (RMSE), the colors indicate spine level as shown in C. **C** Generally, the upper levels had better model fits, and with the addition of more predictors the R^2^ increased and RMSE decreased. An R^2^ ≥ 0.6 and RMSE ≤ 10 years are highlighted green to emphasize well-fit and accurate models, respectively

The regression fits improved from the 1-predictor model (Fig. 4A) to the 4-predictor model (Fig. 4B). The fit lines for all models move closer to the 1:1 line with increasing number of predictors (Fig. 4C). The models produced a good estimate of disc age, with prediction error (RMSE) ≈ 10 years. Across spine levels, L3-L4 had the greatest fit quality (highest R^2^), followed closely by L2-L3, L1-L2, and L4-L5 discs (Fig. 5A). The RMSE ranged from 7–11 years across spine levels, except L5-S1 had greater error (Fig. 5B). With more predictors in the model, the fits and RMSE changed minimally for the upper levels (L1-L4); however, additional predictors improved the model fit in the lower levels L4-S1 (Fig. 5C).

**Fig. 6:**
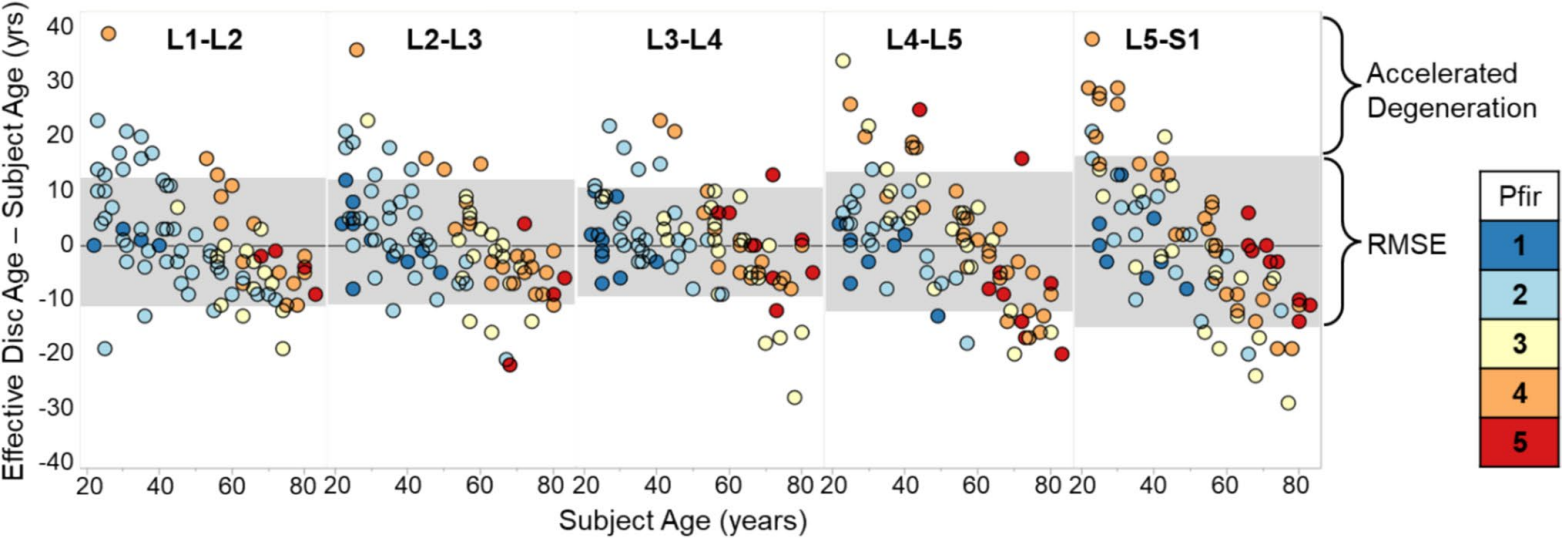
Differences between effective disc age and subject age vs. subject age by spine level. The model can distinguish expected normal aging (gray region) from accelerated degeneration (upper region)

The 4-predictor model was selected as the best model and thus the final effective disc age model, as the 6- or 8-predictor models did not significantly improve R^2^ and RMSE across spine levels. Furthermore, the 4-predictor model includes predictors from all three categories, subject traits, disc geometry, and MRI biomarkers. The intercepts and coefficients for calculating the effective disc age with the 4-predictor model is provided for each spine level for use in future applications (Table 3). Comparison of a disc’s effective age to the subject’s true age provides a simple, objective assessment of normal disc aging (Fig. 6).

**Table 3:** Effective disc age model details for the recommended 4-predictor model with nucleus T2 time (ms), subject height (inches), mid-sagittal disc area (mm^2^) and 3D disc volume (mm^3^)

4-Predictor model	Predictor	L1–L2	L2–L3	L3–L4	L4–L5	L5–S1
Model intercept		1.423 E + 02	1.402 E + 02	1.634 E + 02	1.563 E + 02	1.658 E + 02
Model coefficient	NP T2 time (ms)	− 3.950 E − 01	− 3.879 E − 01	− 3.645 E − 01	− 3.319 E − 01	− 2.701 E − 01
Model coefficient	Subject Ht (in)	− 1.050 E + 00	-8.855 E − 01	− 1.263E + 00	− 1.297E + 00	− 1.498E + 00
Model coefficient	Mid-sagittal disc area (mm^2^)	− 4.852 E − 02	− 7.195 E− 02	− 1.058 E− 01	− 9.644 E − 02	− 1.399 E− 01
Model coefficient	3D disc volume (mm^3^)	2.585 E − 03	2.040 E − 03	2.737 E − 03	2.920 E − 03	4.336 E − 03
R^2^ (goodness of fit)	–	0.68	0.70	0.77	0.62	0.43
RMSE (root mean square error)	–	10.4	10.1	8.8	11.3	13.8

#### Model evaluation

The selected 4-predictor model was further evaluated. Standard beta coefficients were used to measure the contributions of each parameter for each spine level (Table 4). Age estimation for the upper levels, L1-L2 and L2-L3, was primarily driven by the NP T2 time and moderately from the disc volume. Age estimates for L3-L4 and L4-L5 depended on NP T2 time and disc volume nearly equally. Lastly, estimates for L5-S1 were driven primarily by disc area and volume, with moderate contribution from NP T2 time and subject height. The variance inflation factors (VIF) confirmed minimal multicollinearity between predictors (Table 4). The probability values confirmed statistically significant contributions from all predictors across all levels except the L1-L2 disc area (Table 4).

**Table 4:**
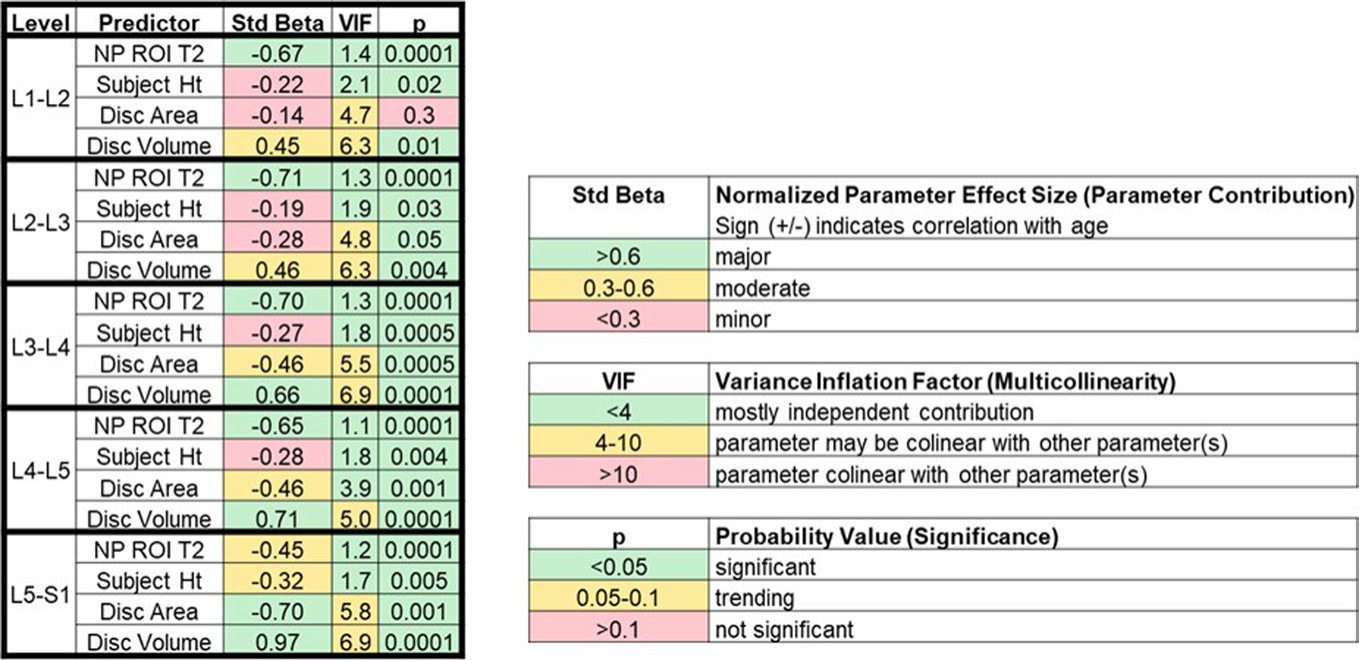
The 4-predictor model including nucleus T2 time (NP ROI T2), subject height, disc area, and disc volume were further assessed. The normalized predictor effect size was evaluated by standard beta (Std Beta), predictor collinearity was evaluated by variance inflation factors (VIF), and predictor significance was evaluated by *p* values (*p*) for the predictor models by spine level

**Fig. 7:**
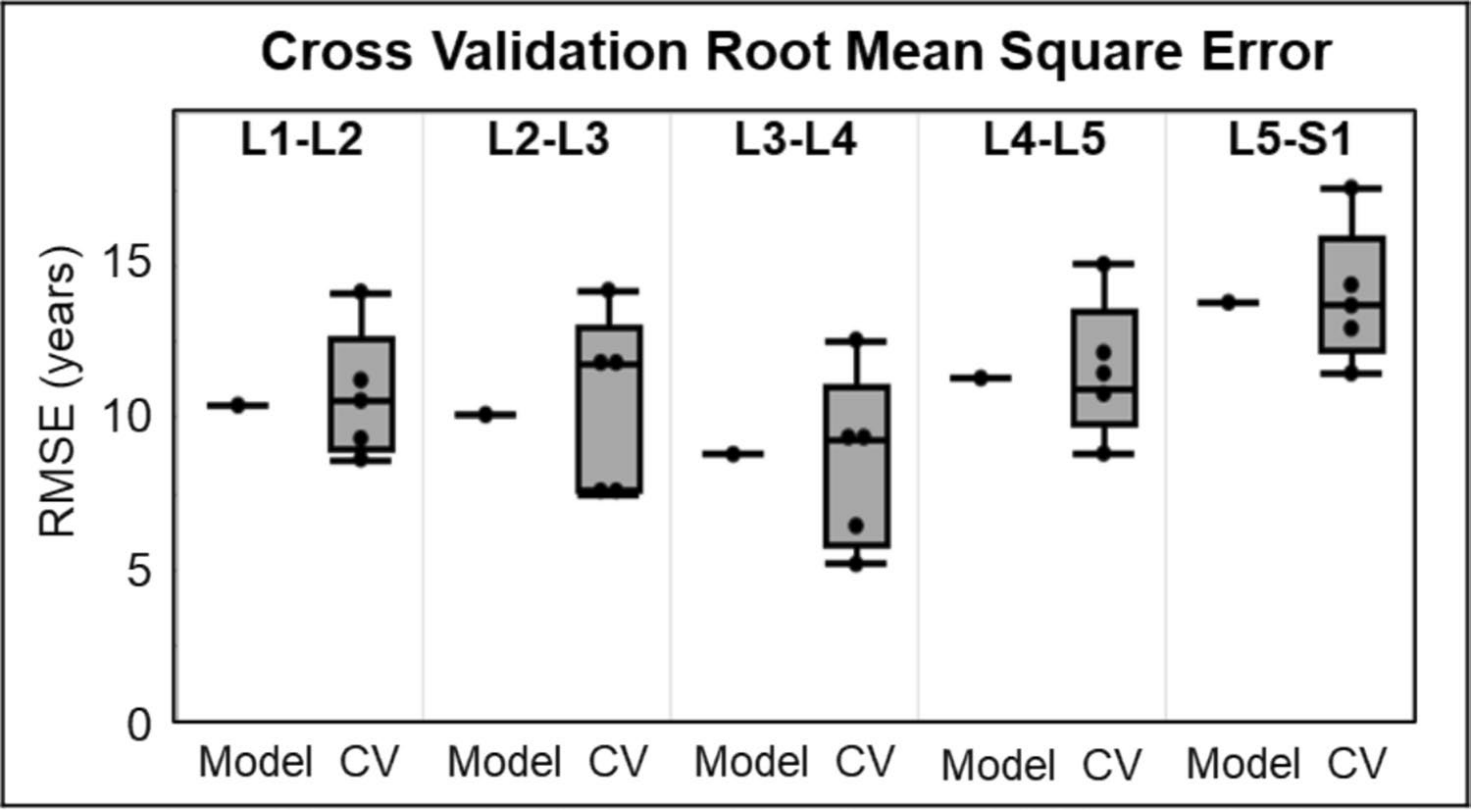
Root mean square error (RMSE) of the “Model” fit quality for the effective age model, fit to all data (n = 84 per spine level) and quantile box-and-whisker plots of the “CV”, fivefold cross validation subsets (80% fit / 20% validation)

Finally, the 4-predictor model was evaluated by fivefold cross validation to quantify model uncertainty and check generalizability. The cross-validation RMSE was offset from the full 4-predictor model by only a few years (Fig. 7). The model uncertainty was ± 3 years, which is less than the RMSE (≈ 10 years), confirming that the model is generalizable, such that subsets of the dataset used to fit the model do not substantially change its accuracy.

## Discussion

This study used a population of 84 healthy, asymptomatic subjects of uniformly distributed age and sex to establish a 4-predictor statistical model for normal aging of the intervertebral disc. This model addresses a key challenge in the field—quantifying normal aging to enable distinction between normal aging and accelerated degeneration [6–8]. Using the 4 predictors of NP T2 time, subject height, disc mid-sagittal area, and disc volume, the model calculates the age at which a disc would, on average, exhibit the observed phenotype; i.e., the “effective disc age”. The difference between the effective disc age and the subject’s actual age provides a simple, objective assessment of degeneration in excess of that expected from normal aging; i.e., “accelerated degeneration” (Fig. 6). The input measurements are easily obtained from MRI. In contrast to qualitative scoring systems, the effective disc age model does not suffer from inter-observer variability or adjacent disc bias, and confidence intervals for the model output can be directly calculated from known measurement uncertainty in the model inputs.

An important advantage in using this model is its ability to control for level-dependency in the presentation of age-associated disc degeneration [8, 9, 11, 23, 24]. This is critically important because recent work has shown controlling for level and age strengthens even the association between nonspecific disc generation (Pfirrmann grade) and low back pain [22]. Furthermore, heritability analysis shows that the majority of genetic and environmental influences on disc degeneration phenotypes are specific to the upper (L1–L4) or lower (L4–S1) lumbar spine [31]. A common quantitative measure of disc degeneration, NP T2 time, has a relationship to Pfirrmann grade that varies by spine level [5, 13, 21]. We similarly found differences between disc levels in the relationships between the considered predictor variables and aging (Table 4; Figs. 5, S2 and S4A). Level-dependency is not accounted for in existing degeneration grading schemes [1, 2, 4], and would be difficult to consider in any qualitative scale, but is easily accounted for by level-specific coefficients in the effective age model (Table 3).

This work provides a data-driven, quantitative tool to distinguish normal aging from other forms of disc degeneration. It does not solve the overarching and persistent problem of specifically identifying pathological degeneration [7], but is nevertheless an important step towards that goal. In our asymptomatic study population, effective age closely tracked true age for spine levels L1-L5 (R^2^ ≈ 0.7, RMSE ≈ 10 years), and moderately so for L5–S1 (R^2^ = 0.43, RMSE = 14 years). These R^2^ values are also similar to the amount of degeneration attributable to genetic influence [7, 31]. Degenerative changes from normal, non-pathological aging in the upper lumbar spine are therefore predictable, constituting the majority of observed variation. Existing grading schemes, which lump normal aging together with all other forms of degeneration, correlate poorly with pathology [9–11, 16–20]. The effective age model, in contrast, allows future research to screen out discs with degeneration typical of normal aging and focus on discs with more interesting, and potentially clinically relevant, degenerative changes. Although we observed some discs classified as having accelerated degeneration (Fig. 6, top band), the number was small, as expected in an asymptomatic population, and may include mere outliers. In future studies with a symptomatic subject pool, we expect to identify more discs with accelerated degeneration.

This work was not without limitations. The effective disc age model does not consider other observable disc characteristics such as Schmorl’s nodes, spondylolisthesis, or Modic changes, though neither do existing grading schemes [1–5]. While an advantage of our study is all scanning was done consistently at 8am, if future studies scan at different times of day, they may require a minor adjustment to account for diurnal changes. Fortunately, these diurnal effects are minor, for example, T2 time changes by ~ 8 ms diurnally [27, 32], which would have a minimal effect on effective disc age ~ 2–3 years, less than the RMSE. Here, the effective disc age was calculated for each disc independently, without reference to trends or patterns across the subject’s whole spine. Prior work has suggested a whole lumbar spine assessment could provide useful context [9]. Finally, the effective age model has not yet been applied to a low back pain population.

In summary, this study established a statistical model of effective disc age to assess disc health based on NP T2 time, subject height, and disc geometry. The model addresses limitations in existing grading schemes, which do not account for age effects or spine level and are qualitative, subjective, and ordinal. The difference between effective disc age and a subjects’ true age distinguishes predictable degeneration, *normal aging* (effective disc age ≈ true age), from potentially *accelerated degeneration* (effective disc age > true age), with the latter being much less common and possibly having a greater likelihood of present or future pathology. The statistical model for effective disc age fills a critical need for an age-dependent degeneration assessment, with the potential to impact many clinical and research applications.

## Supplementary Information

Below is the link to the electronic supplementary material.

Supplementary file1 (DOCX 3718 KB)

## Data Availability

Data will be made available upon request.
