# Supplementary material for: Effective disc age: a statistical model for age-dependent and level-specific lumbar disc degeneration using magnetic resonance imaging (MRI)
Source: Eur Spine J. 2025 Mar 1;34(5):1697–708. doi: 10.1007/s00586-025-08729-9 (PMC12106566; doi:10.1007/s00586-025-08729-9)

**SUPPLEMENTAL**

1. **T2 Line Methods**

Initially, we sought to use a winged trapezoid fit on each individual disc and determine changes in region size with age, but due to the significant changes in T2 time with aging and degeneration, the trapezoidal fit became unreasonable as regions were increasingly indistinguishable with age. Therefore, we instead used the trapezoidal fit to determine the average size of the disc regions in a relatively healthy subset of the discs. The T2 line regions were determined from a subset of discs consisting of only Pfirrmann grade 1 and 2 (n = 186 discs, 44% of all discs in study) which were fit to a winged trapezoid (Figure S1 A-B), such that the first wing was the anterior annulus (AAF), the first sloping region was the anterior transition (ATrans), the plateau was the nucleus pulposus (NP), the second sloping region was the posterior transition (PTrans), and the final wing was the posterior annulus (PAF). From this healthy subset of discs, the average size of each region was determined and then used to evaluate the T2 time of each region for all discs, where the AAF was the first 12%, ATrans was 28%, NP was 34%, PTrans was 17%, and the PAF was the last 9% (Figure S1 C).

Figure S1: A/B) From the T2-w CPMG images, a 3-pixel tall line from the anterior to posterior of the disc was evaluated across 100 points which were then fit with a winged trapezoid (blue). C) The average width of each of the 5-winged trapezoid regions across discs with Pfirrmann grade 1 or 2 were then used as the regional boundaries for all discs to determine comparable regional T2 times in the anterior annulus (AAF), anterior transition (ATrans), nucleus pulposus (NP), posterior transition (Ptrans), and posterior annulus (PAF).


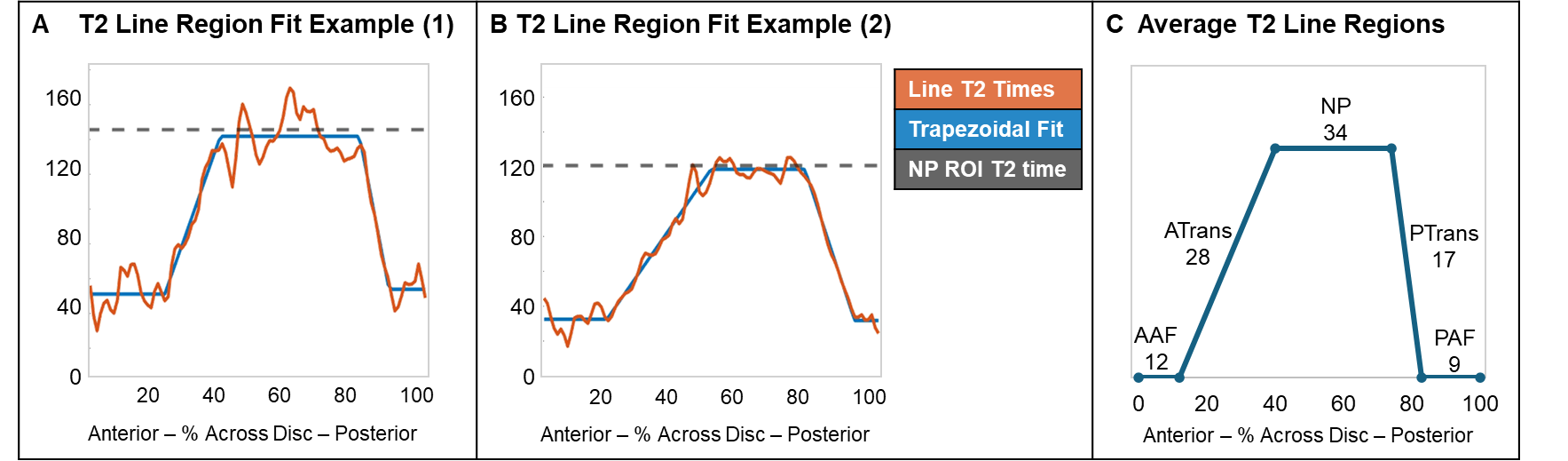


The regional T2 time was therefore ultimately evaluated by a 3-pixel tall line, drawn across the anterior-posterior of the disc in the mid-sagittal slice. The line was subsampled to a normalized 100 intensity values that were then fit with the noise-corrected exponential [25]. The line was then divided into five regions based on the trapezoidal analysis explained above.

1. **MRI Outcomes**
   1. **Disc Geometry by Spine Level**

2D geometry measures for disc mid-sagittal height, anterior-posterior width, and mid-sagittal area all increased inferiorly down the spine as expected (Figure S2 A-C). Disc 3D volume increased inferiorly from the L1-L2 to the L4-L5; however, the L5-S1 was only larger than the L1-L2 (Figure S2 D). Disc wedge angle increased inferiorly, with the L5-S1 having the largest wedge angle on average, and the greatest variability (Figure S2 E). Both the anterior and posterior bulges increased inferiorly with the L5-S1 having the greatest bulge and variability (Figure S2 F-G).

Figure S2: Disc geometry trends by level, moving inferiorly down the spine: A) disc height, B) width, C) area, D) volume, E) wedge, and F,G) bulge generally increase. Across all measures, L5-S1 has the greatest variability and does not always follow the lumbar disc geometry trends. Any levels that do not share a letter are significantly different from each other.


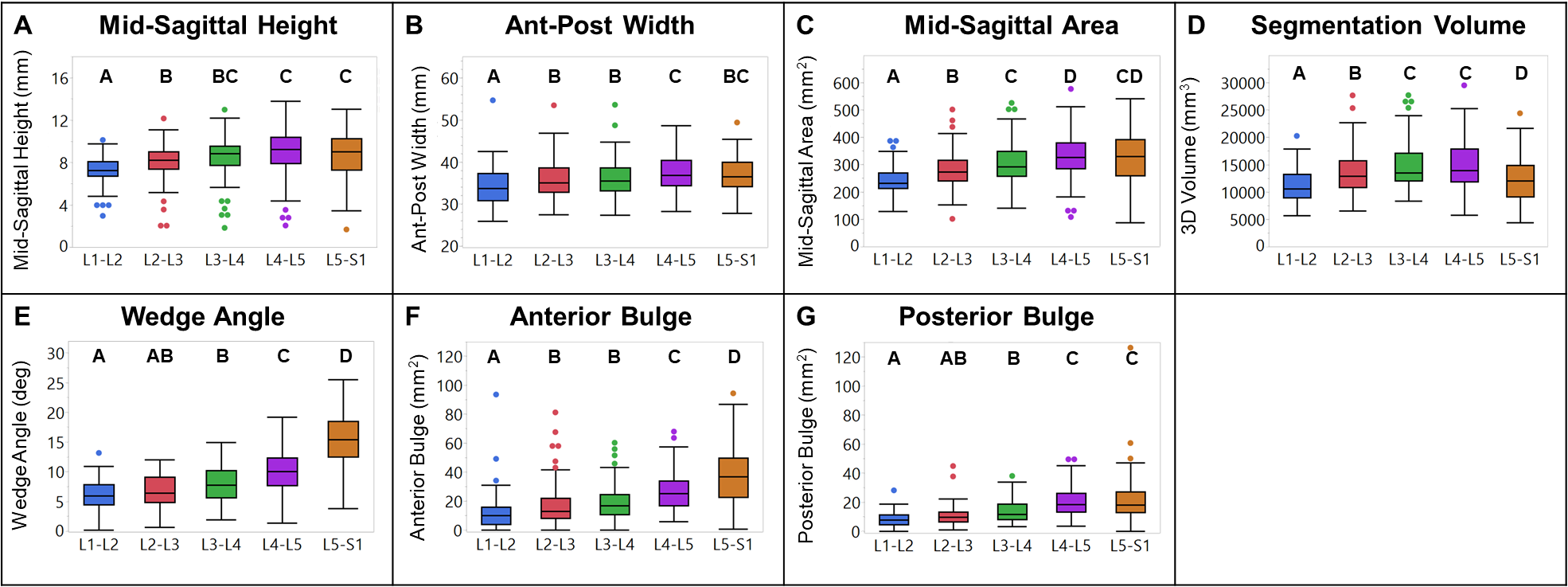


- 1. **Disc Geometry with Age**

With increasing subject age, disc height decreased while width and bulge increased (Figure S3 A-B, F-G). The mid-sagittal disc area did not change significantly with age, such that the reduction in height and increase in width negated each other in the mid-sagittal slice (Figure S3 C). However, across the 3D disc volume there was a slight increase with age (Figure S3 D). The disc wedge angle was not significantly correlated with age (Figure S3 E). The reduction of proteoglycans and water in the NP reduced the NP pressure, allowing disc height to decrease and subsequently reducing the tension in the annulus. The reduced annulus tension and breakdown of collagen and matrix collectively contribute to the increase in width and bulges with age.

Figure S3: Disc geometry trends with increasing subject age: A) disc height decreases, B) width increases, C) midsagittal area is unchanged, D) disc volume increases slightly, E) wedge angle is unchanged and F,G) bulge increases.


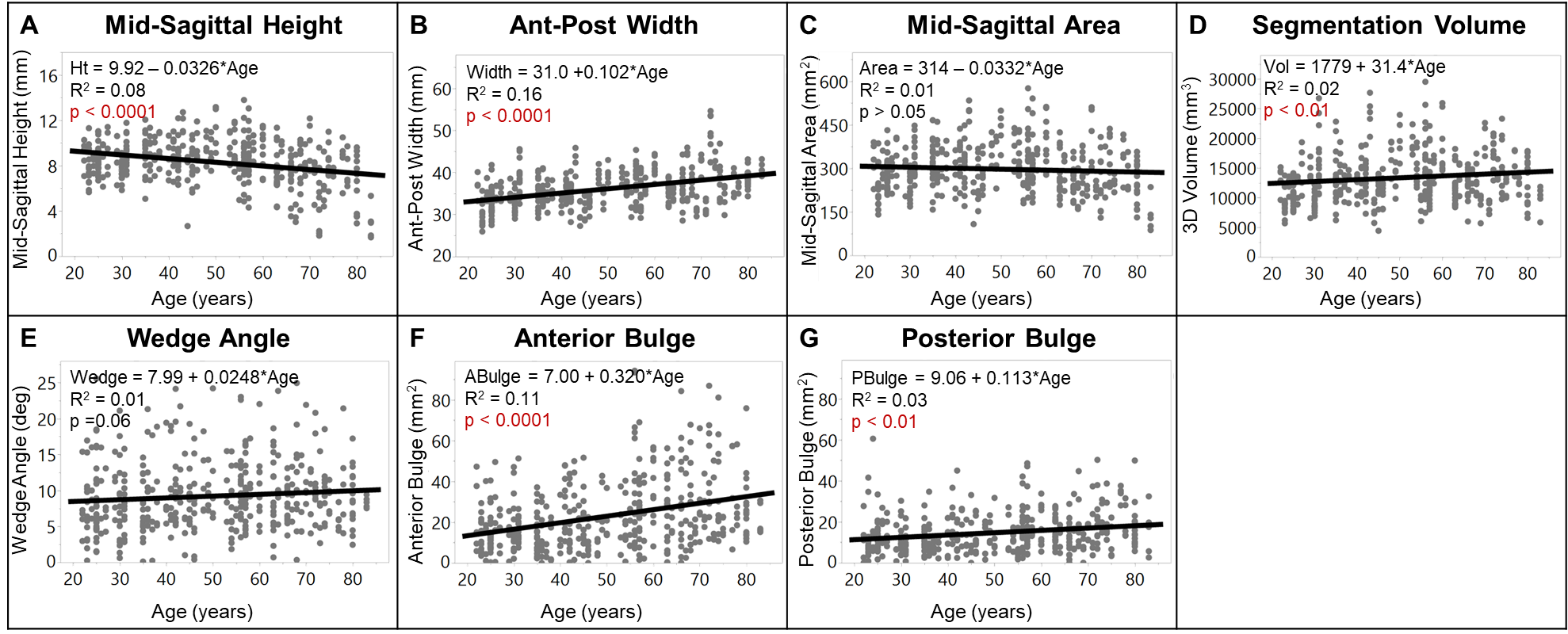


- 1. **T2 Relaxation Time**

In addition to disc geometry, T2 relaxation time, a biomarker for disc composition and degeneration was assessed in several regions by spine level and with age. The T2 time measured from the NP region of interest (ROI) decreased slightly from the upper to lower spine levels (Figure S4 A). There was similar variation and spread of T2 times across all levels. The distinction between regions by T2 time is most evident in younger subjects, the NP T2 time decreases significantly from the 18-39 year old groups to the 40-59 year old groups to the 60+ year old groups (Figure S4 B-C, Figure S5). The NP T2 time decrease with age is greater than the AF T2 time increases with age, such that distinguishing regions by T2 time is increasingly difficult with increasing age (Figures S4 D, Figure S5).

Figure S4: A) T2 relaxation time in the nucleus pulposus (NP) decreased inferiorly down the spine. B) From the T2 line regions, the NP T2 time was significantly greater than the transition and annulus fibrosus (AF) regions. The posterior AF was also greater than the anterior AF. C) Young subjects had higher NP T2 times and lower AF T2 times compared to older subjects. D) The NP T2 time decreased with age and AF T2 times increased with age. For subfigures A and B, any levels that do not share a letter are significantly different from each other.


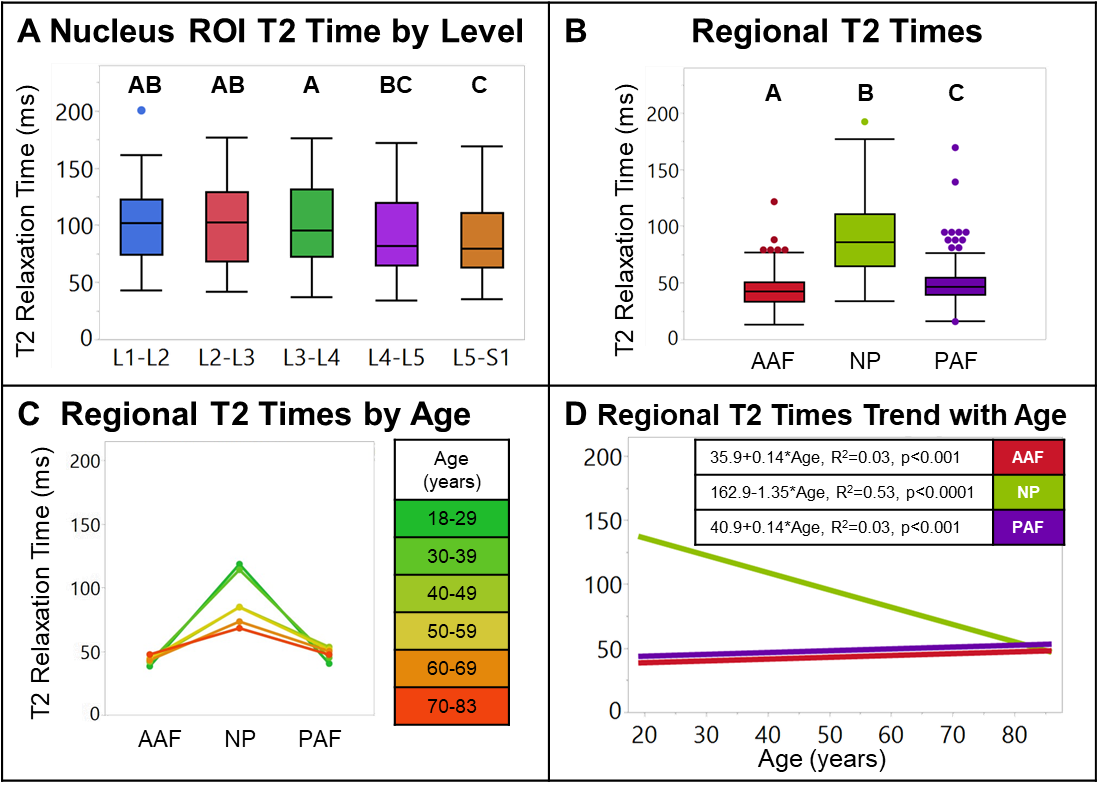


Figure S5: A,B) T2 relaxation time in the NP decreases significantly with age while the C) anterior and D) posterior AF T2 time increases with age. The NP T2 time calculated from the A) ROI was consistently greater than the NP T2 time from the B) line analysis by 5ms ± 18ms (p<0.0001).


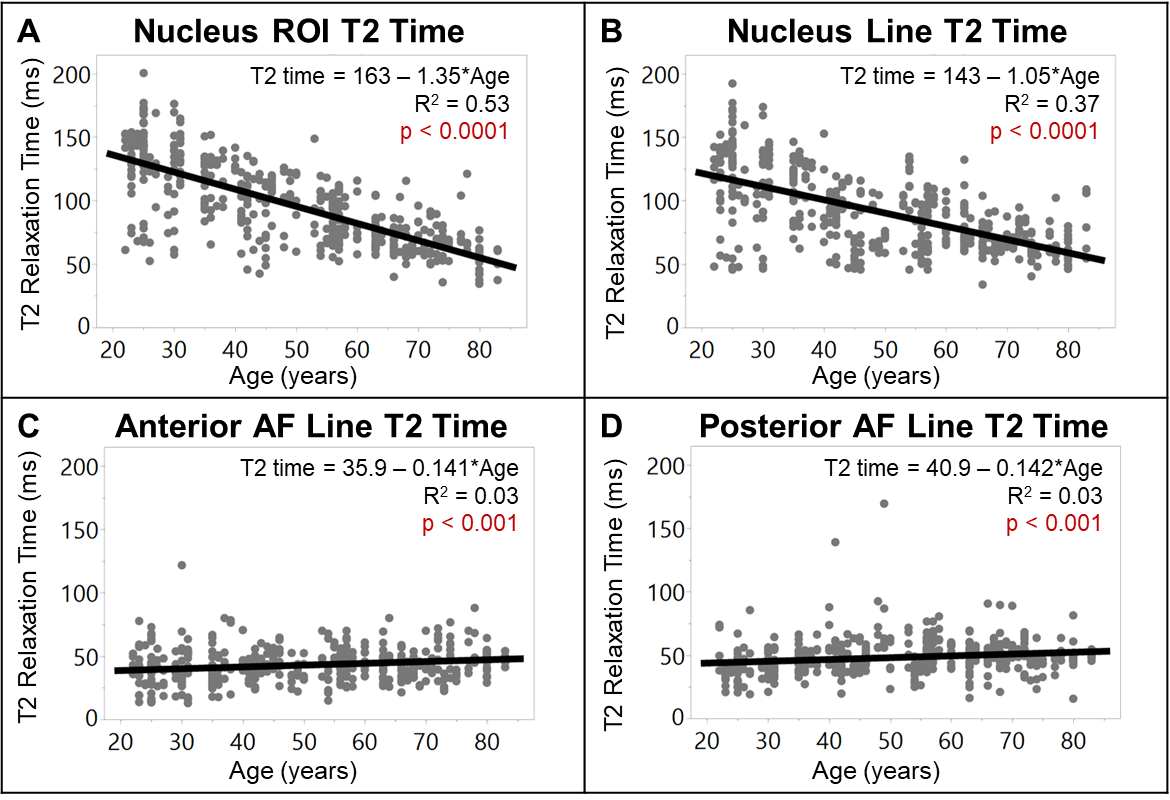


- 1. **Disc Features**

Disc features including Schmorl’s nodes, collapsed discs, spondylolisthesis, and sacralized L5-S1 were recorded by spine level and age decade. A total of 420 discs were evaluated with 84 discs per level and 70 discs per decade, the features are summarized in Table S1.

Table S1: Summary of disc features recorded by spine level and age decade. A total of 420 discs were evaluated with 84 discs per level and 70 discs per decade. Several discs had multiple features recorded such that the total number/percent of discs by level or decade may exceed the expected total.


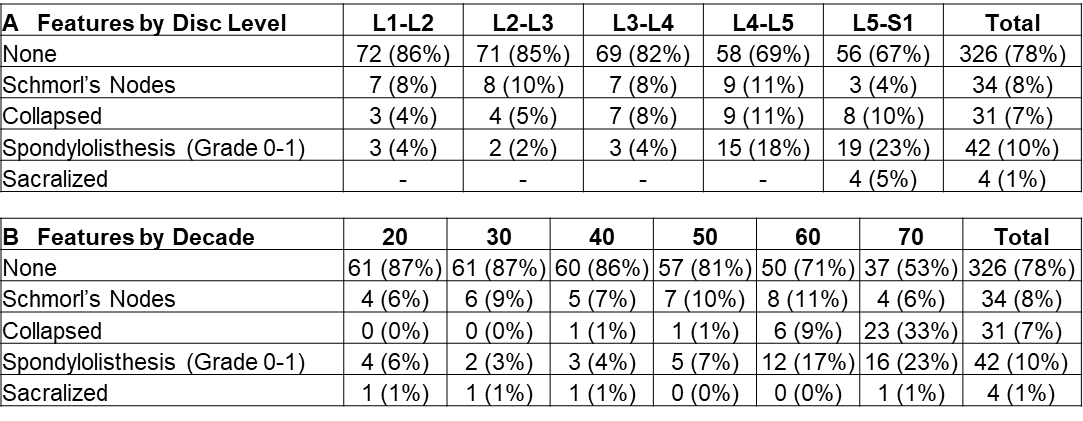

Supplement: Supplementary file 1 — Supplementary file1 (DOCX 3718 KB) [file 586_2025_8729_MOESM1_ESM.docx]
